# Epidemiology of physical inactivity in Nigeria: a systematic review and meta-analysis

**DOI:** 10.1093/pubmed/fdab147

**Published:** 2021-05-13

**Authors:** Davies Adeloye, Janet O Ige-Elegbede, Asa Auta, Boni M Ale, Nnenna Ezeigwe, Chiamaka Omoyele, Mary T Dewan, Rex G Mpazanje, Emmanuel Agogo, Wondimagegnehu Alemu, Muktar A Gadanya, Michael O Harhay, Akindele O Adebiyi

**Affiliations:** Centre for Global Health, Usher Institute, University of Edinburgh, Edinburgh, UK; Centre for Public Health and Wellbeing, University of the West of England, Bristol, UK; School of Pharmacy and Biomedical Sciences, University of Central Lancashire, Preston, UK; Holo Healthcare Limited, Nairobi, Kenya; Federal Ministry of Health, Abuja, Nigeria; Federal Ministry of Health, Abuja, Nigeria; World Health Organization, Nigeria Country Office, Abuja, Nigeria; World Health Organization, Nigeria Country Office, Abuja, Nigeria; Resolve to Save Lives, Abuja, Nigeria; International Health Consultancy, LLC, Atlanta, GA, USA; Department of Community Medicine, Aminu Kano Teaching Hospital, Bayero University, Kano, Nigeria; Department of Biostatistics, Epidemiology and Informatics, University of Pennsylvania Perelman School of Medicine, Philadelphia, PA, USA; College of Medicine, University of Ibadan, Ibadan, Nigeria

**Keywords:** physical inactivity, prevalence, noncommunicable diseases, risk, epidemiology, Nigeria

## Abstract

**Background:**

Physical activity is crucial to preventing noncommunicable diseases. This study aimed to provide up-to-date evidence on the epidemiology of insufficient physical activity across Nigeria to increase awareness and prompt relevant policy and public health response.

**Methods:**

A systematic literature search of community-based studies on physical inactivity was conducted. We constructed a meta-regression epidemiologic model to determine the age-adjusted prevalence and number of physically inactive persons in Nigeria for 1995 and 2020.

**Results:**

Fifteen studies covering a population of 13 814 adults met our selection criteria. The pooled crude prevalence of physically inactive persons in Nigeria was 52.0% (95% CI: 33.7–70.4), with prevalence in women higher at 55.8% (95% CI: 29.4–82.3) compared to men at 49.3% (95% CI: 24.7–73.9). Across settings, prevalence of physically inactive persons was significantly higher among urban dwellers (56.8%, 35.3–78.4) compared to rural dwellers (18.9%, 11.9–49.8). Among persons aged 20–79 years, the total number of physically inactive persons increased from 14.4 million to 48.6 million between 1995 and 2020, equivalent to a 240% increase over the 25-year period.

**Conclusions:**

A comprehensive and robust strategy that addresses occupational policies, town planning, awareness and information, and sociocultural and contextual issues is crucial to improving physical activity levels in Nigeria.

## Introduction

Physical inactivity has been described as a global pandemic, partly responsible for the rising burden of noncommunicable diseases (NCDs) across world regions.^1^ In 2015, physical inactivity directly contributed to 21% of breast cancers, 25% of colon cancers, 27% of diabetes and 30% of ischemic heart diseases globally.^2^ In sub-Saharan African, the World Health Organization (WHO) estimated about three million physical inactivity-related deaths in 2014.^3^ The benefits of staying physically active have been well documented; nonetheless, about a quarter of the global adult population do not currently meet the WHO recommendations of staying active (i.e. 150 minutes of moderate physical activity or 75 minutes of vigorous physical activity per week).^3^ The WHO estimates that annual global deaths from physical inactivity are currently above three million.^3^^,^^4^ African countries, including Nigeria, bear disproportionately higher burden, partly due to fast rising urbanization and economic growth, with consequent increase in unhealthy lifestyles and sedentary living across many settings.^5^

In Nigeria, the barriers to being physically active appear to be mediated by a couple of contextual factors.^6^ Rapid urbanization and widespread industrial activities in the country have created several environmental challenges that affect healthy behaviors across many Nigerian cities.^7^ High density traffic, poor road designs and unsafe terrains characterize many cities, with recreational walking and cycling unappealing to many.^8^ The prevailing low levels of health literacy and sociocultural barriers are additional contextual issues. Many regard cycling or walking as a sign of a low socioeconomic status, hence would rather prefer to own and/or drive a car for better societal recognition and respect.^8^^,^^9^

Nigeria currently has a population of over 200 million, which is the highest in Africa and possibly includes the highest population of physical inactive persons on the continent. Indeed, the prevalence of physical inactivity is reportedly high, ranging from 25% to 57%, with this linked to higher prevalence rates of obesity, type 2 diabetes and cancer.^6^^,^^10^ However, the epidemiology of physical inactivity in the country is still poorly understood. There is limited data from many settings, no nation-wide report, and obviously no national policy and population response. It therefore becomes imperative to coalesce available data to provide the evidence needed to effect relevant public health policies, changes and reforms in the country. We conducted a comprehensive and systematic search of publicly available sources in Nigeria to provide nation-wide and regional estimates of the prevalence of physical inactivity in the country.

## Methods

### Search strategy

We searched relevant databases, including MEDLINE, EMBASE, Global Health and Africa Journals Online (AJOL), for studies on physical inactivity in Nigeria. Search terms are shown in Table 1. Searches were conducted on 30 July 2020 and limited to studies published after 1 January 1990. Unpublished documents were sourced from Google Scholar and Google searches. Titles and abstracts of studies were reviewed, and full-texts of relevant studies accessed. The reference lists of accessed full-texts were further hand-searched for additional studies. We contacted authors of selected papers for any missing information.

**Table 1:** Search terms on physical inactivity in Nigeria

**#**	*Searches*
1	africa/or africa, sub-sahara/or africa, western/or nigeria/
2	exp vital statistics/
3	(incidence* or prevalence* or morbidity or mortality).tw.
4	(disease adj3 burden).tw.
5	exp "cost of illness"/
6	case fatality rate.tw
7	hospital admissions.tw
8	Disability adjusted life years.mp.
9	(initial adj2 burden).tw.
10	exp risk factors/
11	2 or 3 or 4 or 5 or 6 or 7 or 8 or 9 or 10
12	exp physical inactivity/or physical inactive/or sedentary lifestyles
13	1 and 11 and 12
14	Limit 13 to “1990-current”

### Selection criteria

We selected population-based studies reporting on the prevalence of physical inactivity in a Nigerian setting among persons aged 15 years or more. However, due to high number of studies on cardiometabolic risks identified from an initial scoping exercise and reporting physical inactivity, we also carefully reviewed several studies on NCDs risks in Nigeria and extracted data on physical inactivity from such studies when reported. We excluded hospital-based reports, studies on Nigerians in diaspora, reviews, view-points and commentaries.

### Case definitions

Currently, the WHO recommends that adults aged between 18 and 64 years should engage in at least 150 minutes of moderate-intensity aerobic exercise per week, or at least 75 minutes of vigorous-intensity aerobic exercise throughout the week, or a combination of both.^4^ We broadly regarded persons not meeting this definition as physically inactive, or having insufficient or inadequate physical activity. However, this definition was not applied across all studies, with some defining physical activity according to previous guidelines (30 minutes of moderate physical activity per day for at least 5 days in a week).^11^ Moreover, some studies assessed physical activity based on work or household activities, transport related activities, farming, walking, running, climbing or other moderate to vigorous activities, with timing varying from 20 to 30 minutes per day. Thus, there were varying definitions employed by different surveys on physical inactivity in Nigeria. However, to ensure some level of consistency in case ascertainment, we checked if the study participants were evaluated using the WHO STEPwise approach to Surveillance (STEPS) of NCDs protocol or a modified version.^12^ We considered the process of case ascertainment as one of the criteria in determining the overall quality of each study (see Quality assessment).

**Table 2:** Quality assessment of selected studies

*Quality criteria*	*Assessment*	*Score*	*Maximum score*
Sampling method (was it representative of a target subnational population?)	Yes	1	1
	No	0	
Appropriateness of statistical analysis	Yes	1	1
	No	0	
Case ascertainment (was it based on standard or modified WHO STEPS criteria, unspecified criteria, informal interviews, or not reported?)	Standard WHO STEPS	3	3
	Unspecified criteria	2	
	Informal interviews	1	
	Not reported	0	
Total (high (4–5), moderate (2–3) or low quality (0–1))			5

### Data extraction

Assessment of eligible studies was conducted independently by two reviewers (DA, JOI and AA), with an eligibility guideline to ensure consistency in study selection and extraction. Disagreements were resolved by consensus. Data on the location, study period, study design, study setting (urban or rural), sample size, diagnostic criteria and mean age of the population were extracted. These were matched with corresponding data on physically inactive persons, sample population, prevalence of physical inactivity in each study. For studies conducted on the same study site, population or cohort, the first published study was selected, and all additional data from the other studies were extracted and merged with data from the selected paper.

### Quality assessment

For each full text selected, DA and JOI further screened for explicit description of methodology, case definitions, and generalizability of reported estimates to a larger population within the geopolitical zone. For the quality grading, we adapted a previously used quality assessment guideline for studies examining the prevalence of chronic diseases.^13^ For each full text selected, we screened for (i) sampling strategy (was it representative of a target subnational population, for example, local government area or town population where the study was conducted), (ii) statistical methods (was it appropriate for the study outcome?) and (iii) case ascertainment (was it based on standard WHO STEPS criteria, unspecified criteria, informal interviews, or not reported?). Studies were graded as ‘high’ (4–5), ‘moderate’ (2–3) or ‘low quality’ (0–1) (see Tables 2 and 3 and Supplementary Material**,** for details of all full-text manuscripts accessed and quality grading).

**Table 3:** Characteristics of studies on prevalence of physical inactivity in Nigeria

*Author*	*Study period*	*Location*	*Geopolitical zone*	*Study setting*	*Quality grade*
Agaba *et al.*^18^	2014	Jos, Plateau State	North–central	Urban	High
Emerole *et al*.^19^	2007	Owerri, Imo State	South–east	Urban	Moderate
Odugbemi *et al.*^20^	2010	Tejuosho, Lagos	South–west	Urban	Moderate
Ige *et al.*^21^	2013	Ibadan, Oyo State	South–west	Urban	High
Ugwuja *et al.*^32^	2008	Abakaliki, Ebonyi State	South–east	Urban	Moderate
Oladapo *et al.*^22^	2000	Egbeda, Oyo State	South–west	Rural	Moderate
Odenigbo *et al.*^23^	2008	Asaba, Delta State	South–south	Semiurban	Moderate
Adegoke and Oyeyemi^24^	2011	Ibadan, Oyo State	South–west	Semiurban	High
Oyeyemi and Adeyemi^25^	2013	Maiduguri, Borno State	North–east	Semiurban	Moderate
Odunaiya *et al.*^26^	2010	Ibadan, Oyo State	South–west	Urban	Moderate
Owoeye *et al.*^27^	2013	Lagos State	South–west	Urban	High
Oyeyemi *et al.*^28^	2013	Maiduguri, Borno State	North–east	Semiurban	Moderate
Ezejimofor *et al.*^29^	2014	Niger Delta, Delta State	South–south	Rural	Moderate
Ezekwesili *et al.*^30^	2016	Anambra State	South–east	Mixed Urban–Rural	Moderate
Ogah *et al.*^31^	2012	Umuahia, Abia State	South–east	Mixed Urban–Rural	High

### Data analysis

A random effects meta-analysis, using the DerSimonian and Laird Method,^14^ was employed on the individual study estimates to pool crude national and subnational summary estimates of the prevalence of physical inactivity in Nigeria. Standard errors were determined from the reported crude estimates and population denominators, based on a binomial (or Poisson) distribution. Heterogeneity between studies was assessed using I-squared (*I^2^*) statistics, and subgroup analysis was further conducted to detect causes of heterogeneity. A meta-regression epidemiologic model accounting for study sample size, study period and age was constructed to determine prevalence distribution of physical inactivity by age of the Nigerian population. We employed the model to estimate the absolute number of physically inactive persons in Nigeria at midpoints of the United Nation (UN) population 5-year age groups for Nigeria for the years 1995 and 2020.^15^ Our approach to data analysis has been described in detail in previous studies.^13^^,^^16^ All statistical analyses were conducted on STATA (Stata Corp V.14, TX, USA). The study was conducted according to the PRISMA guidelines,^17^ and all data employed in the study are provided in the Supplementary Material.

## Results

### Search results

**Fig. 1:**
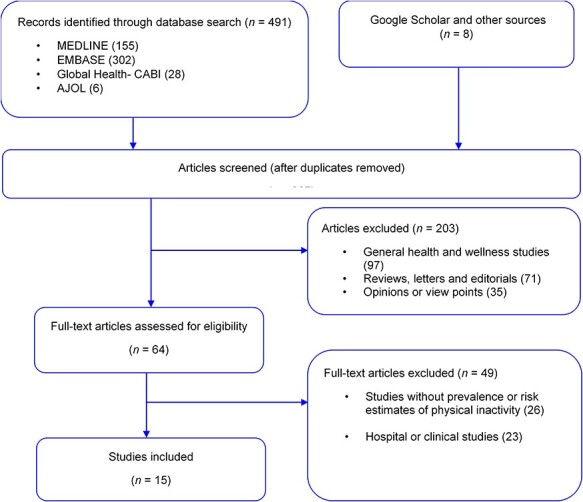
Flow chart of selection of studies on physical inactivity in Nigeria.

Our searches returned 491 articles from the databases (MEDLINE 155, EMBASE 302, Global Health 28 and AJOL 6). Additional eight studies were identified through Google Scholar, and hand-searching reference lists of relevant studies. After duplicates have been removed, 267 titles were screened for relevance (i.e. any population-based studies on physical inactivity in Nigeria). On applying the selection criteria, 203 studies were excluded. Sixty-four full-texts were assessed and screened explicitly using the selection and quality criteria. Fifteen articles^18–32^ were selected for the review (Fig. 1).

### Study characteristics

The 15 studies were selected across the southern and northern parts of Nigeria (Table 3). Six studies were retained from the South–west, four from South–east, two each from South–south and North–east and one from the North–central. Seven studies were conducted in urban settings, six in semiurban settings or among a mix of urban and rural dwellers and two in rural settings. Five studies were rated as high quality, with 10 rated as moderate quality. Study period ranged from 1995 to 2016, with most studies conducted within a 1-year period. The total population from all studies was 13 814, with aggregated mean age ranging from 22 to 53 years (Table 3). Heterogeneity was high across studies, with I-squared (*I^2^*) estimated at 99.0% (*P* < 0.001). When the geopolitical zones were considered as individual subgroups, heterogeneity was highest in the South–west, at 99.9%.

### Crude prevalence of physical inactivity in Nigeria

**Fig. 2:**
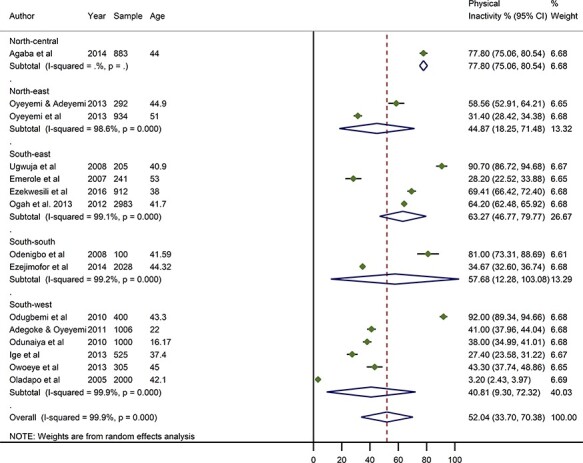
Crude prevalence rate of physical inactivity in Nigeria, by geopolitical zones.

**Fig. 3:**
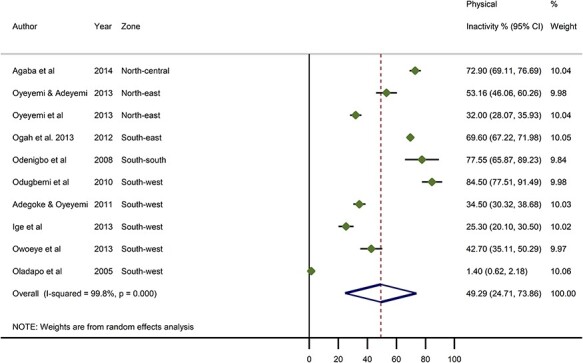
Crude prevalence rate of physical inactivity in Nigeria, men.

**Fig. 4:**
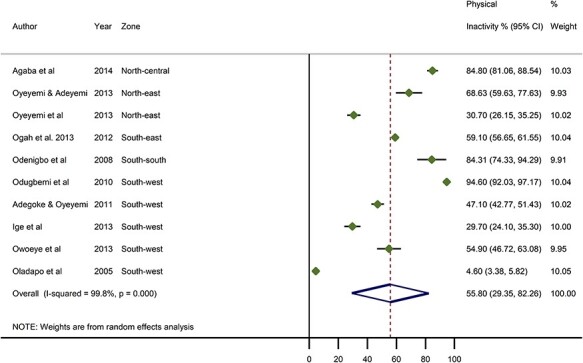
Crude prevalence rate of physical inactivity in Nigeria, women.

From all studies, the highest prevalence of physical inactivity was reported among traders in an urban market in Lagos State, South–west Nigeria at 92%.^20^ Other equally high estimates of physically inactive persons were reported among civil servants in Abakaliki, Ebonyi State, South–east Nigeria and among middle-class professionals in Asaba, Delta State, South–south Nigeria, both at 91% and 81%, respectively.^23^^,^^32^ The lowest prevalence of physically inactive persons was reported among rural dwellers in Egbeda, Oyo State, South–west Nigeria, at 3.2%.^22^ From all data points, the pooled crude prevalence of physically inactive persons in Nigeria was 52.0% (95% CI: 33.7–70.4) (Fig. 2). From a sensitivity analysis, we estimated a pooled crude prevalence of 50.8% (95% CI: 34.1–67.5) from only high-quality studies, which is statistically not different from the overall pooled estimate (Supplementary Material). The prevalence in women was higher at 55.8% (95% CI: 29.4–82.3) compared to men at 49.3% (95% CI: 24.7–73.9) (Figs. 3 and 4**,**Table 4). The prevalence was highest in North–central (77.8%, 75.1–80.5), followed by South–east (63.3%, 46.8–79.8) and South–south (57.7%, 12.3–93.1). The North–east (44.9%, 18.3–71.5) and South–west (40.8%, 9.3–72.3) had lowest estimated prevalence. As observed in the distribution of prevalence rates reported by individual studies, prevalence of physically inactive persons was significantly higher in urban settings (56.8%, 35.3–78.4) compared to rural settings (18.9%, 11.9–49.8) (Table 4, Supplementary Material).

### Estimated number of physically inactive persons in Nigeria

The meta-regression epidemiologic model, adjusted for study period and sample size (total 13 814), was applied to mean ages and crude prevalence rates of physical inactivity extracted from all studies. When absolute cases were estimated, we observed an increasing prevalence with age. Using the UN demographic projections for Nigeria, we estimated about 14.4 million physically inactive persons in Nigeria in 1995 among persons aged 20–79 years. Driven partly by the rapid demographic changes observed in Nigeria, this increased significantly to over 48.6 million physically inactive persons among persons aged 20–79 years in 2020. During this 25-year period, the age-adjusted prevalence of physical inactivity in Nigeria doubled from 29% to 58%, with absolute number of physically inactive persons aged 20–79 years increasing by about 240% (Table 5).

**Table 4:** Pooled crude estimates of prevalence of physical inactivity in Nigeria

Region		Both sexes		Men		Women	
		*Prevalence % (95% CI)*	*I^2^, P-value*	*Prevalence % (95% CI)*	*I^2^, P-value*	*Prevalence % (95% CI)*	*I^2^, P-value*
Nation-wide	*All studies*	52.0 (33.7–70.4); 50.8 (34.1–67.5)*	99.0, 0.000	49.3 (24.7–73.9)	99.8, 0.000	55.8 (29.4–82.3)	99.8, 0.000
Geopolitical zone	*North–central*	77.8 (75.1–80.5)	-	-	-	-	-
	*North–east*	44.9 (18.3–71.5)	98.6, 0.000	-	-	-	-
	*South–east*	63.3 (46.8–79.8)	99.1, 0.000	-	-	-	-
	*South–south*	57.7 (12.3–93.1)	99.2, 0.000	-	-	-	-
	*South–west*	40.8 (9.3–72.3)	99.9, 0.000	-	-	-	-
Settings	*Urban*	56.8 (35.3–78.4)	99.6, 0.000	-	-	-	-
	*Rural*	18.9 (11.9–49.8)	99.9, 0.000	-	-	-	-
	*Mixed*	57.4 (43.8–71.1)	99.1, 0.000	-	-	-	-

^*^High-quality studies.

**Table 5:** Absolute number of physical inactive persons aged 20 years or more in Nigeria, 1995 and 2020

Age (years)	1995			2020		
	*Prevalence (%)*	*Population (000)*	*Cases (000)*	*Prevalence (%)*	*Population (000)*	*Cases (000)*
20–24	26.9	9732.072	2616.078	55.861	15981.820	8927.604
25–29	27.5	7814.716	2152.642	56.526	14051.044	7942.493
30–34	28.211	6586.947	1858.244	57.191	12102.265	6921.406
35–39	28.876	5534.292	1598.082	57.856	9982.646	5775.560
40–44	29.541	4611.630	1362.322	58.521	7767.685	4545.727
45–49	30.206	3894.188	1176.278	59.186	6008.701	3556.310
50–54	30.871	3330.832	1028.261	59.851	4993.836	2988.861
55–59	31.536	2690.877	848.595	60.516	4146.148	2509.083
60–64	32.201	2090.951	673.307	61.181	3325.733	2034.717
65–69	32.866	1544.460	507.602	61.846	2554.200	1579.671
70–74	33.531	1031.795	345.971	62.511	1821.521	1138.651
75–79	34.196	581.547	198.866	63.176	1077.611	680.792
**All (20–79)**	**29.055**	**49444.307**	**14366.248**	**57.987**	**83813.210**	**48600.874**

Note: Estimates based on the epidemiologic modeling from all data points.

## Discussion

### Main findings of this study

Our study broadly suggests about 50 million persons in Nigeria do not engage in sufficient physical activity on a weekly basis in 2020, using the WHO reference, representing an age-adjusted prevalence of 58%. When the regions were considered, the South–west had the lowest prevalence of physical inactivity in Nigeria at 40.8%, although this appears to be due to larger number of studies conducted in rural settings. When compared with the prevalence in the South–east and South–south regions, we observed higher prevalence patterns at 63.3% and 57.7%, respectively. This could be as a result of widespread sedentary occupational patterns across several urban settings in Southern Nigeria.^13^ This corroborates a significantly higher prevalence of physical inactive persons estimated among urban dwellers compared to rural dwellers, already well documented in previous studies.^7^^,^^10^ It appears an increasing rural–urban migration in Nigeria and emergence of highly congested urban slums with poor designs for leisure activities, sidewalks, running and cycling are leading factors for this considerable geographic difference. Meanwhile, there is very limited data to describe the prevalence pattern of physical inactivity in Northern Nigeria. Historically, the Northern parts of Nigeria have large groups of nomadic herdsmen who travel long distances daily,^16^ which possibly reflects the relatively lower prevalence of physically inactive persons in the North–east. Although the high rate estimated among university employees in the North–central suggests a need for further studies in this region.

Meanwhile, we reported a higher prevalence of physical inactivity among women at 55.8% compared to men at 49.3%. This appears to be a familiar trend in many African settings. The pooled prevalence of physical inactivity across 22 African countries among women was 24%, whereas men recorded a prevalence of 16%.^33^ Several reasons have been reported by different authors. For example, African American women have described personal care after exercise as major barriers, noting that perspiration from physical activity affects hairstyles and appearance, and restyling tends to be time-consuming and expensive.^34^ Besides, sociocultural, religious and traditional norms attached to women in many African settings are leading reasons why they less engage in outdoor aerobic exercises.^33^ Differences in occupational patterns, with men engaged in physically demanding jobs and women in domestic or sedentary jobs, are other important considerations.^35^

### What is already known

Findings from this review are in keeping with some earlier reports. In 2020, we estimated a total of 48.6 million physically inactive persons in Nigeria, accounting for an age-adjusted prevalence of 58%. This estimate is congruent with the range provided by Abubakari and Bhopal,^10^ who reported that 25–57% of Nigerians are physically inactive. This is also in the range of the estimates reported in some neighboring African countries. According to Guthold *et al.*,^33^ the prevalence of physically inactive persons in Mali, Cote d’Ivoire and Cameroon were 58.2%, 41.8%, and 41.7%, respectively. A 2018 global study^1^ estimated that the prevalence of insufficient physical activity in Nigeria ranged was 27.1% (21.5–33.5), although this was mainly conducted in metropolitan Maiduguri, North-eastern Nigeria. This, nonetheless, still falls within the lower confidence interval of our estimate (33%). Moreover, in the same study,^1^ the overall prevalence of insufficient physical activity in sub-Saharan Africa was relatively low at 21.4%, ranging from 5·5% in Uganda to 41.3% in Mauritania. Asides poor health literacy, sociocultural practices, high traffic density, poor road designs and other challenges linked to rapid urbanization, increasing crime and security challenges in several parts of Nigeria also imply that many would avoid early morning or late evening outdoor aerobic exercises. This possibly explains the higher rates estimated in Nigeria compared to other African settings.

### What this study adds

This review, through a detailed systematic search, has identified important community-based studies on physical inactivity in Nigeria. It provides up-to-date estimates of zonal and national prevalence of physically inactive persons in Nigeria and how these vary across age groups, gender, over a 25-year period. To the best of our knowledge, this is the first comprehensive nation-wide estimate of the prevalence of physical inactivity in Nigeria.

Our findings have important public health and policy implications. Although we observed a 240% increase in the number of persons with insufficient physical activity in Nigeria between 1995 and 2020, population-wide measures to address physical activity levels are largely unavailable. This appears to be a global issue, as the WHO also reported that the global target to reduce physical inactivity by 10% by 2025 has been slow and off track, suggesting an urgent implementation or scale-up of effective policies across member countries.^5^ Although, the Nigerian Federal Ministry of Health drafted a strategic plan to tackle major risk factors of NCDs in 2013, implementation has not been as expected, particularly for physical activity.^36^ Specifically, there is a need for occupational policies that incorporate some level of activities into work schedules across many urban settings. In addition, a review of national and regional town planning guidelines is crucial to ensuring outdoor physical activities are accessible and safe for all.^4^ It is also important that information and awareness campaigns on physical activity and health address relevant contextual and sociocultural issues in different settings, particularly among women.

Moreover, the high levels of physical inactivity found in this study have important implications for an increasing national burden of NCDs—a major risk for COVID-19 mortality. Indeed, it is reasonable to assume that the COVID-19 pandemic, with the lockdowns, social-distancing restrictions and increasing virtual meetings, would further increase the already high levels of physical inactivity in the country.^37^ This highlights a need to raise awareness on the importance of indoor aerobic exercise during the pandemic to improve overall health and wellbeing, and possibly reduce individual risk factors that could result in COVID-19 related complications and deaths.

### Limitations of this study

Our limitations are also important study findings. First, we retrieved only three articles from Northern Nigeria, with none from the North–west. This challenge has been documented in previous national studies on NCDs.^13^^,^^16^ Indeed, the understanding of the burden and trend of NCDs risks across Nigeria has been limited by few original population-based studies, particularly from the Northern parts of the country. Including hospital-based studies, that could offer additional insights, would make pooling rather inappropriate, and in fact, contribute to an already high heterogeneity. Besides, denominators for hospital catchments were not reported in many studies, which further justify reasons for exclusion. Second, the quality of research on physical inactivity appears to be generally low. This may be, in part, due to poor reporting and/or the stringent requirement of our quality assessment tools. The varying study designs and case definitions employed across studies, some of which were not in line with standard survey protocols for estimating NCDs risks, further contributed to the overall low quality of research. This, in addition to other individual and population differences across selected studies, accounted for the high heterogeneity in our data. Although our sensitivity analysis (based on the five high-quality studies) revealed a prevalence of 50.8% (**S**upplementary Material), which is relatively close to our overall pooled crude estimate of 52.0%; we emphasize caution in the interpretation of our age-adjusted estimates as our model have been based on few reported individual figures and extrapolation of scarce data. Strengthening surveillance and research capacities will be vital for future nation-wide research efforts,^38^ and an important complementary measure to providing relevant interventions when needed.

## Conclusions

Our study suggests a high and increasing prevalence of physically inactive persons in Nigeria. There are still widespread gaps in the overall response across the country, including research, policy and interventions. A comprehensive and robust strategy that addresses occupational policies, town planning, awareness and information, and sociocultural and contextual issues is crucial to improving physical activity levels across the country.

## Supplementary Material

Physical_Nig_Supplementary_fdab147

## Notes

**Davies Adeloye**, MD, PhD

**Janet O Ige-Elegbede**, MSc

**Asa Auta**, PhD

**Boni M Ale**, MD

**Nnenna Ezeigwe**, MD

**Chiamaka Omoyele**, MSc

**Mary T Dewan**, MD

**Rex G Mpazanje**, MD

**Emmanuel Agogo**, MD

**Wondimagegnehu Alemu**, MD

**Muktar A Gadanya**, MD

**Michael O Harhay**, PhD

**Akindele O Adebiyi**, MD, PhD
