# Supplementary material for: Epidemiology of physical inactivity in Nigeria: a systematic review and meta-analysis
Source: J Public Health (Oxf). 2021 May 13;44(3):595–605. doi: 10.1093/pubmed/fdab147 (PMC9424057; doi:10.1093/pubmed/fdab147)
Supplement: Physical_Nig_Supplementary_fdab147 [file physical_nig_supplementary_fdab147.docx]

**SUPPLEMENTARY FILE**

**Epidemiology of physical inactivity in Nigeria: a systematic review and meta-analysis**

**Davies Adeloye^1*∞^, Janet O Ige-Elegbede^2∞^, Asa Auta****^3^, Boni M Ale^4^, Nnenna Ezeigwe^5^, Chiamaka Omoyele^5^, Mary T Dewan^6^, Rex G Mpazanje^6^, Emmanuel Agogo^7^, Wondimagegnehu Alemu^8^, Muktar A Gadanya^9^, Michael O Harhay^10^, Akindele O Adebiyi^11^**

1. Centre for Global Health, Usher Institute, University of Edinburgh, UK.
2. Centre for Public Health and Wellbeing, University of the West of England, Bristol, UK.
3. School of Pharmacy and Biomedical Sciences, University of Central Lancashire, Fylde Road, Preston, UK
4. Holo Healthcare Limited, Nairobi, Kenya
5. Federal Ministry of Health, Abuja, Nigeria
6. World Health Organization, Nigeria Country Office, Abuja, Nigeria
7. Resolve to Save Lives, Abuja, Nigeria
8. International Health Consultancy, LLC, Atlanta, Georgia, USA
9. Department of Community Medicine, Aminu Kano Teaching Hospital, Bayero University, Kano, Nigeria
10. Department of Biostatistics, Epidemiology and Informatics, University of Pennsylvania Perelman School of Medicine, Philadelphia, Pennsylvania, USA
11. College of Medicine, University of Ibadan, Ibadan, Nigeria

∞Contributed equally

**^*^****Correspondence: Dr. Davies Adeloye,** Centre for Global Health, Usher Institute, University of Edinburgh, UK. Email: davies.adeloye@ed.ac.uk

**Figure S1. Crude prevalence of physical inactivity in Nigeria, from high quality studies only.**

**Figure S2. Crude prevalence of physical inactivity in Nigeria, by study settings.**

**Table S1. Quality appraisal**

| Author | Sampling | Analysis | Ascertainment | Score | Quality grade |
| --- | --- | --- | --- | --- | --- |
| Agaba et al (1) | 1 | 1 | 2 | 4 | High |
| Emerole et al (2) | 1 | 1 | 1 | 3 | Moderate |
| Odugbemi et al (3) | 1 | 1 | 1 | 3 | Moderate |
| Ige et al (4) | 1 | 1 | 2 | 4 | High |
| Ugwuja et al (5) | 1 | 1 | 1 | 3 | Moderate |
| Oladapo et al (6) | 1 | 1 | 1 | 3 | Moderate |
| Odenigbo et al (7) | 1 | 1 | 1 | 3 | Moderate |
| Adegoke & Oyeyemi (8) | 1 | 1 | 2 | 4 | High |
| Oyeyemi & Adeyemi (9) | 1 | 1 | 1 | 3 | Moderate |
| Odunaiya et al (10) | 1 | 1 | 1 | 3 | Moderate |
| Owoeye et al (11) | 1 | 1 | 2 | 4 | High |
| Oyeyemi et al (12) | 1 | 1 | 1 | 3 | Moderate |
| Ezejimofor et al (13) | 1 | 1 | 1 | 3 | Moderate |
| Ezekwesili et al (14) | 1 | 1 | 1 | 3 | Moderate |
| Ogah et al (15) | 1 | 1 | 2 | 4 | High |

**Table S2. All extracted data employed in analysis**

| Study ID | Author | Study Period | Location | Geopolitical zone | Study design | Study Setting | Population | Lifestyle type | Definition | Mean age | Cases (all) | Sample (all) | Prev % (all) | Age (male) | Cases (male) | Sample (male) | Prev % (male) | Age (female) | Cases (female) | Sample (female) | Prev % (female) |
| --- | --- | --- | --- | --- | --- | --- | --- | --- | --- | --- | --- | --- | --- | --- | --- | --- | --- | --- | --- | --- | --- |
| 9 | Agaba et al | 2014 | Jos, Plateau State | North-central | Descriptive cross-sectional study | Urban | Academic institution | **Physical inactivity** |  | 44 | 687 | 883 | 77.80 | 43 | 386 | 529 | 72.90 | 45 | 300 | 354 | 84.80 |
| 23 | Emerole et al | 2007 | Owerri, Imo State | South-east | Descriptive cross-sectional study | Urban | Higher education institution | **Physical inactivity** | No moderate recrational or cocupational exertion | 53 | 68 | 241 | 28.20 |  |  |  |  |  |  |  |  |
| 33 | Odugbemi et al | 2010 | Tejuosho, Lagos | South-west | Descriptive cross-sectional study | Urban | Traders | **Physical inactivity** | Engaging in exercise lasting less than 30 minutes per day and for less than 5 days a week | 43.3 | 368 | 400 | 92.00 | 45.5 | 87 | 103 | 84.50 | 42.3 | 281 | 297 | 94.60 |
| 35 | Ige et al | 2013 | Ibadan, Oyo State | South-west | Descriptive cross-sectional study | Urban | Higher education institution | **Physical inactivity** | Lack of regular, sustained activity i.e. when the total physical activity was below the recommended (i.e. <30 minutes of moderate activity per day for at least 3 days/week). | 37.4 | 144 | 525 | 27.40 | 37.4 | 68 | 269 | 25.30 | 37.4 | 76 | 256 | 29.70 |
| 36 | Ugwuja et al | 2008 | Abakaliki, Ebonyi State | South-east | Descriptive cross-sectional study | Urban | Civil servants | **Physical inactivity** |  | 40.9 | 186 | 205 | 90.70 | 40.9 |  | 106 |  | 40.9 |  | 99 |  |
| 39 | Oladapo et al | 2005 | Egbeda, Oyo State | South-west | Descriptive cross-sectional study | **Rural** | General population | **Physical inactivity** |  | 42.1 | 64 | 2000 | 3.20 | 42.1 | 12 | 873 | 1.40 | 42.1 | 52 | 1127 | 4.60 |
| 68 | Odenigbo et al | 2008 | Asaba, Delta State | South-south | Population-based cross-sectional study | Semi-urban | General population | **Physical inactivity** |  | 41.59 | 81 | 100 | 81.00 | 41.59 | 38 | 49 | 77.55 | 41.59 | 43 | 51 | 84.31 |
| 69 | Adegoke & Oyeyemi | 2011 | Ibadan, Oyo State | South-west | Descriptive cross-sectional study | Semi-urban | Higher education institution | **Physical inactivity** |  | 22 | 412 | 1006 | 41.00 | 22 | 171 | 496 | 34.50 | 22 | 240 | 510 | 47.10 |
| 72 | Oyeyemi & Adeyemi | 2013 | Maiduguri, Borno State | North-east | Population-based cross-sectional study | Semi-urban | General population | **Physical inactivity** | moderate to high sedentary lifestyles | 44.9 | 171 | 292 | 58.56 | 44.9 | 101 | 190 | 53.16 | 44.9 | 70 | 102 | 68.63 |
| 76 | Odunaiya et al | 2010 | Ibadan, Oyo State | South-west | Population-based cross-sectional study | Urban | Secondary school | **Physical inactivity** | Low physical activity | 16.17 | 380 | 1000 | 38.00 |  |  |  |  |  |  |  |  |
| 78 | Owoeye et al | 2013 | Lagos State | South-west | Descriptive cross-sectional study |  | Civil servants | **Physical inactivity** | Global Physical Activity Questionnaire (GPAQ) | 45 | 132 | 305 | 43.30 | 45 | 70 | 163 | 42.70 | 45 | 78 | 142 | 54.90 |
| 79 | Oyeyemi et al | 2013 | Maiduguri, Borni State | North-east | Population-based cross-sectional study | Semi-urban | General population | **Physical inactivity** |  | 51 | 293 | 934 | 31.40 | 51 | 173 | 540 | 32.00 | 51 | 121 | 394 | 30.70 |
| 86 | Ezejimofor et al | 2014 | Niger Delta, Delta State | South-south | Community-based cross-sectional study | Rural | General population | **Physical inactivity** |  | 44.32 | 703 | 2028 | 34.67 | 44.32 |  | 871 |  | 44.32 |  | 1157 |  |
| 105 | Ezekwesili et al | 2016 | Anambra State | South-east | Population-based cross-sectional study | Mixed | General population | **Physical inactivity** | moderate to high sedentary lifestyles | 38 | 633 | 912 | 69.41 |  |  |  |  |  |  |  |  |
| 112 | Ogah et al. 2013 | 2012 | Umuahia, Abia State | South-east | Population-based cross-sectional study | Mixed | General population | **Physical inactivity** |  | 41.7 | 1914 | 2983 | 64.2 | 41.5 | 995 | 1430 | 69.6 | 41.8 | 918 | 1553 | 59.1 |

**Table S3. Metaregression**

**Retained studies**

1. Agaba EI, Akanbi MO, Agaba PA, Ocheke AN, Gimba ZM, Daniyam S, et al. A survey of non-communicable diseases and their risk factors among university employees: a single institutional study. Cardiovascular journal of Africa. 2017;28(6):377-84.

2. Emerole CO, Aguwa EN, Onwasigwe CN, Nwakoby BA. Cardiac risk indices of staff of Federal University Of Technology Owerri, Imo State, Nigeria. Tanzania health research bulletin. 2007;9(2):132-5.

3. Odugbemi TO, Onajole AT, Osibogun AO. Prevalence of cardiovascular risk factors amongst traders in an urban market in Lagos, Nigeria. The Nigerian postgraduate medical journal. 2012;19(1):1-6.

4. Ige OK, Owoaje ET, Adebiyi OA. Non communicable disease and risky behaviour in an urban university community Nigeria. African health sciences. 2013;13(1):62-7.

5. Ugwuja E, Ogbonna N, Nwibo A, Onimawo I. Overweight and Obesity, Lipid Profile and Atherogenic Indices among Civil Servants in Abakaliki, South Eastern Nigeria. Annals of medical and health sciences research. 2013;3(1):13-8.

6. Oladapo OO, Salako L, Sodiq O, Shoyinka K, Adedapo K, Falase AO. A prevalence of cardiometabolic risk factors among a rural Yoruba south-western Nigerian population: a population-based survey. Cardiovascular journal of Africa. 2010;21(1):26-31.

7. Odenigbo CU, Oguejiofor OC, Odenigbo UM, Ibeh CC, Ajaero CN, Odike MA. Prevalence of dyslipidaemia in apparently healthy professionals in Asaba, South South Nigeria. Nigerian journal of clinical practice. 2008;11(4):330-5.

8. Adegoke BO, Oyeyemi AL. Physical inactivity in Nigerian young adults: prevalence and socio-demographic correlates. Journal of physical activity & health. 2011;8(8):1135-42.

9. Oyeyemi AL, Adeyemi O. Relationship of physical activity to cardiovascular risk factors in an urban population of Nigerian adults. Archives of public health = Archives belges de sante publique. 2013;71(1):6.

10. Odunaiya NA, Ayodele OA, Oguntibeju OO. Physical activity levels of senior secondary school students in Ibadan, western Nigeria. The West Indian medical journal. 2010;59(5):529-34.

11. Owoeye OB, Osho OA, Akinfeleye AM, Akinsola OJ, Durowoju OS, Akinbo SR. Physical activity profile of senior civil servants in Lagos, Nigeria: need for effective strategies for improvement. The Nigerian postgraduate medical journal. 2013;20(2):104-7.

12. Oyeyemi AY, Usman MA, Oyeyemi AL, Jaiyeola OA. Casual blood pressure of adolescents attending public secondary schools in Maiduguri, Nigeria. Clinical hypertension. 2015;21:16.

13. Ezejimofor MC, Uthman OA, Maduka O, Ezeabasili AC, Onwuchekwa AC, Ezejimofor BC, et al. The Burden of Hypertension in an Oil- and Gas-Polluted Environment: A Comparative Cross-Sectional Study. American journal of hypertension. 2016;29(8):925-33.

14. Ezekwesili CN, Ononamadu CJ, Onyeukwu OF, Mefoh NC. Epidemiological survey of hypertension in Anambra state, Nigeria. Nigerian journal of clinical practice. 2016;19(5):659-67.

15. Ogah OS, Madukwe OO, Chukwuonye, II, Onyeonoro UU, Ukegbu AU, Akhimien MO, et al. Prevalence and determinants of hypertension in Abia State Nigeria: results from the Abia State Non-Communicable Diseases and Cardiovascular Risk Factors Survey. Ethnicity & disease. 2013;23(2):161-7.
